# OVERVIEW OF THE ACTIVE STUDY AT 20 YEARS

**DOI:** 10.1093/geroni/igz038.1616

**Published:** 2019-11-08

**Authors:** Sherry Willis, George Rebok

**Affiliations:** 1 University of Washington, Seattle, Washington, United States; 2 Johns Hopkins Bloomberg School of Public Health, Baltimore, Maryland, United States

## Abstract

ACTIVE (1998-2019), the largest NIA-funded clinical trial of cognitively normal elderly, was designed to test if cognitive intervention maintains functional independence in older adults (N = 2,802, aged 65-94) by improving basic mental abilities. In this paper we overview major aims of ACTIVE to investigate 1) effectiveness and durability (through 1,2,3,5, and 10 years of follow-up) of three cognitive interventions (memory, reasoning, processing speed) in improving basic measures of cognition; 2) if training in specific cognitive abilities improves or maintains cognitively demanding daily living skills (e.g., medication use, driving); and 3) impact of intervention on everyday mobility, health-related quality of life, and health service utilization. We also review aims of a recent NIA-funded 20-year follow-up of ACTIVE to examine whether improved cognition and daily function results in long-term reduction in dementia risk, years of disability, health care utilization and costs, and increased active years of life in advanced old age.

